# InsectSound1000 An insect sound dataset for deep learning based acoustic insect recognition

**DOI:** 10.1038/s41597-024-03301-4

**Published:** 2024-05-09

**Authors:** Jelto Branding, Dieter von Hörsten, Elias Böckmann, Jens Karl Wegener, Eberhard Hartung

**Affiliations:** 1 Julius Kühn Institute (JKI), Institute for Application Techniques in Plant Protection, Braunschweig, 38104 Germany; 2 Julius Kühn Institute (JKI), Institute for Plant Protection in Horticulture and Urban Green, Braunschweig, 38104 Germany; 3 Christian-Albrechts-Universität zu Kiel, Institute of Agricultural Process Engineering, Kiel, 24118 Germany

**Keywords:** Entomology, Agriculture, Scientific data, Computer science

## Abstract

InsectSound1000 is a dataset comprising more than 169000 labelled sound samples of 12 insects. The insect sound level spans from very loud (*Bombus terrestris*) to inaudible to human ears (*Aphidoletes aphidimyza*). The samples were extracted from more than 1000 h of recordings made in an anechoic box with a four-channel low-noise measurement microphone array. Each sample is a four-channel wave-file of 2500 kHz length, at 16 kHz sample rate and 32 bit resolution. Acoustic insect recognition holds great potential to form the basis of a digital insect sensor. Such sensors are desperately needed to automate pest monitoring and ecological monitoring. With its significant size and high-quality recordings, InsectSound1000 can be used to train data-hungry deep learning models. Used to pretrain models, it can also be leveraged to enable the development of acoustic insect recognition systems on different hardware or for different insects. Further, the methodology employed to create the dataset is presented in detail to allow for the extension of the published dataset.

## Background & Summary

The aim of this paper is to present an extensive dataset of high-quality insect recordings and the reproducible and scalable method used to create them. Furthermore, the method development process is reported to ensure that the presented dataset can be extended in future work. InsectSound1000 was created in a project aimed at designing a digital aid system to support greenhouse growers in pest management. Insect sensor solutions are crucial to such digital systems and are an active and still very challenging research field^1,2^. Acoustics was among the potential sensor solutions explored.

A review of related sensing tasks showed that fields employing deep learning techniques for pattern recognition have achieved significant success. A well-known example is the recent success of bird song recognition systems^3^ (https://birdnet.cornell.edu/). However, the precondition for employing deep learning is the availability of adequate datasets^4^. The currently available insect sound data neither met the requirements regarding quantity or quality nor included insects relevant to plant protection in greenhouses^5^ (http://entnemdept.ufl.edu/Walker/buzz/) (https://www.tierstimmenarchiv.de). Therefore, the primary objective of this project was to create and share such a well-suited insect sound dataset to enable a thorough assessment of the potential of acoustic insect detection solutions.

A preliminary version of the InsectSound1000 dataset was included in a publication focused on developing an acoustic insect detection algorithm robust to environmental noise^6^. The version contained only five of the insects in the complete dataset. This work compared the potential of a single-channel, spectrogram-based classification model to that of a multichannel raw audio model. Both models were trained and tested using a unique environmental noise simulation that mixes insect sounds with background noise recordings made in a greenhouse. Using the simulation, the models were pretrained and tested on clean sound data. By gradually mixing louder noise sounds and repeating training on the altered datasets, the models were adapted to address real-world background noise.

The results showed great potential within acoustic insect detection, as both models could correctly classify more than 85% of the clean insect sounds. Testing with simulated background noise revealed the challenges in practical applications of acoustic detection systems. The results also indicated the benefits of recording and processing multichannel data. While the single-channel spectrogram model essentially failed to recognise any but the loudest sounds in conditions simulating the use of the microphone system inside a greenhouse, the multichannel raw audio model was able to correctly classify 57% of the insect sounds in these conditions by utilising spatial filtering techniques^6^.

The five insects selected for the first study represented a wide range of sound levels and had few similarities in size and body structure. Future studies should include other insects in the dataset. This approach could allow, e.g., an evaluation of recognition potential regarding the biological degree of kinship. Future studies could gain further insight into the potential information contained in insect sounds by evaluating the detection accuracy under laboratory conditions. Moreover, the most likely use case for InsectSound1000 is that of a pretraining dataset. While image recognition models are routinely pretrained on freely available datasets (e.g., ImageNet and COCO), such pretraining material is missing for insect sound recognition. This lack of pretraining data is critical, as a previous study showed that pretraining on laboratory recordings turned out to be the precondition for training models robust to environmental noise^6^.

Although it is unlikely that many subsequent studies will employ the same recording hardware, pretraining on InsectSound1000 should improve model performance in projects using different hardware and insects. With its ample size, InsectSound1000 should provide new models with a means of learning the basic patterns of sound recognition in general and the subtleties of insect sound recognition in particular. Successful pretraining can drastically reduce the demand for training data in subsequent fine-tuning training^7^.

The digitalisation of pest management remains an unsolved task in agriculture involving insects^8^. Apart from applications in pest management, acoustic monitoring has high potential for the realisation of large-scale ecological surveys^2^.

## Methods

Creating an insect sound dataset presents a unique challenge because manually labelling the recordings is impossible. This is especially true considering that the recording equipment used allows for recording sounds that are inaudible and, therefore, unknown to human ears. Consequently, an anechoic box was designed to shield the recording area from environmental noise, bypassing the labelling problem. By placing only insects of one species inside the anechoic box at a time, all sounds recorded in one session should be from that insect species.

Not only are datasets of sufficient size needed, but the creation process must be well documented, reproducible and, ultimately, scalable^9^ so that increasingly more classes can be added to the dataset, increasing its practical relevance. These preconditions set high demands regarding the method of data generation. Therefore, this section describes the recording setup and its development process. In this way, future research in this field will benefit from the findings.

The methods section of this paper is split into three parts. First, the recording setup development is presented. Second, the details of the actual recording process are described. Third, the steps of digital data processing used to create the sound sample dataset from the raw recordings are explained.

### Acoustic recording setup

The development of the recording setup is presented in a structure resembling the steps of an engineering design process. Initially, a short literature review on the methods used in related projects is provided. Building on these findings, the design goals are defined. The next section describes and implements the design choices made in the method development for InsectSound1000.

### Overview of methods used in related research

While reviewing related work, it quickly became apparent that the recordings would need to be made in a noise-shielded environment to bypass the labelling problem. For this reason, the review of related methods is further split into two parts: first, an examination of anechoic boxes used in related research to shield the recording environment from noise and, second, a short review of recording equipment used to record insects in other projects.

**A summary of anechoic boxes built for related research** is provided in Table 1. The summary provides an overview of different published solutions to the problem of noise shielding a small recording environment and their technical details. The first three designs were presented for recording storage pest sounds, while the other designs were proposed in other sound research fields.

**Table 1:** Summary of anechoic box designs used in related research projects.

Source	Layers	Total Weight	Special Solutions	Achieved Sound Absorption
^29^	Single wall: 1. 19 mm plywood 2. 3.2 mm loaded vinyl 3. 76.2 mm foam 4. 3.2 mm loaded vinyl 5. 6.4 mm plywood 6. 152.4 mm foam wedges	81 kg		50 dB to 70 dB between 1 kHz and 10 kHz
^12^	Single wall, cylindrical: 1. 1 mm steel wall 2. 50.8 mm foam wedges 3. different types of foam vinyl, aluminium and lead used to build an entering funnel and a lid	70 kg including the pedestal	4 outside microphones to monitor outside noises	60 dB to 90 dB between 1 kHz and 10 kHz
^13^	Double-wall: 1. 18 mm plywood 2. 50 mm of acoustic foam 3. 50 mm air gap 4. 18 mm plywood 5. three different density foams compressed to 2/3 of their thickness 6. 18 mm plywood	Unknown	Inside box suspended from springs inside the outside box	Not tested
^10^	Double-wall: 1. 30 mm MDF-Board 2. 50 mm air gap filled with glass wool 3. 30 mm MDF-Board 4. 150 mm acoustic wedges, with 30 mm base	220 kg just the inside box	Double-deflection neoprene mounts to mount the inside box inside the outer box	More than 50 dB as low as 200 Hz (Simulation)
^11^	Double-wall: 1. 30 mm MDF-board 2. air gap 3. 30 mm MDF-Board 4. 300 mm acoustic wedges	Unknown		Not tested
^14^	Double-wall: 1. Concrete 2. Foam 3. Light concrete 4. 700 mm acoustic wedges, with 300 mm base	387,000 kg	Entire box placed on springs isolators	More than 50 dB as low as 100 Hz (Simulation)

A comparison of the different designs and materials reveals that only the first two solutions employ a single wall design (Table 1). These sandwich wall designs achieve most of their sound absorption using heavy materials. In physics, this is described by the mass law. This law describes the sound absorption of a material layer in dB as a linearly increasing function over the frequency range. More dense materials will lead to greater sound absorption at lower frequencies. This finding can be observed in Table 1 by comparing the weights and sound absorption of the different designs.

All other designs that were reviewed employed a double-wall setup. Double walls allow for achieving sound absorption greater than that predicted by the mass law by minimising the transmission of sounds between the two walls. This allows double-wall designs to use lighter and less expensive materials while achieving comparable noise reduction levels. Ressl *et al*. presented the theory behind their design in detail^10^. The authors explain their choice of wall thickness and air gap dimension by deliberately designing it so that the first and second resonance frequencies of the double-wall system are placed at the lower and upper bounds of their target frequency range. At these resonance frequencies, standing waves can form between the two walls, causing them to acoustically act as if there was no air gap between them. Therefore, the acoustic isolation of a double-wall structure is greatly reduced at these resonance frequencies. García Rodríguez presented similar calculations^11^.

Ressl *et al*. choose their box dimensions as a prime number multiple of a common scale^10^ to avoid overlapping standing wave modes of the different room dimensions in the relevant frequency range. By avoiding the overlap of the resonance frequencies of the different room modes, the diminishing effect of the resonance frequencies on the overall noise shielding performance can be mitigated.

The special solutions column of Table 1 highlights some interesting ideas from the different designs. These ideas surpass the usual choice of design materials and dimensions. Mankin *et al*. proposed using four external microphones to monitor outside noise during the acoustic evaluation of the grain samples investigated in the study^12^. If the outside noise level rises above a level that corrupts the measurements inside the enclosure, the test recordings would automatically be interrupted.

The use of double-wall structures raises the issue of fixing the two walls relative to each other in a way that minimises sound leakage from the outside wall to the inside wall. While Ressl *et al*. used double-deflection neoprene mounts^10^, Njoroge *et al*. suspended the first box inside the second box from springs^13^, providing a simple, low-cost solution to improve isolation against very low-frequency vibrations travelling through the building structures.

The solution proposed by Rusz showcases the possible performance if very heavy and expensive materials are used^14^. The simulation results for the 387 t heavy chamber show that the proposed rugged design should provide a sound transmission loss above 50 dB for frequencies as low as 100 Hz.

**A summary of the recording equipment used in related research** is provided in Table 2. This study provides an overview of different published solutions to the problem of selecting recording equipment suitable for recording very low-level insect sounds. This literature review indicates that a broad range of microphones of different qualities and costs have been utilised to record insect sounds for research purposes.

**Table 2:** Summary of recording equipment used in related research.

Source	Target Species and Sound	Microphone Type	Microphone Sensitivity	Data Acquisition	Recording Environment
^15^	calling song of fruit flies Anastrepha ludens	Consumer Microphone (System MZK 80ZU, Sennheiser)	Unknown	digital camera recorder	Petri dish
^13^	storage pests in maize grain, movement and feeding sounds	1/2” standard measurement microphone (378B02, PCB Piezotronics Inc.)	50 mV/Pa	16 bit A-D-converter (imc C-SERIES, CS-3008-N, imc Meßsysteme GmbH)	Anechoic Box
^16^	walking sounds of different insects on different substrates	1/2” low noise measurement microphone (type 40HH, G.R.A.S.)	800 mV/Pa	custom-built external A/D converter, 16 bit	50x50cm plastic arenas filled with e.g. sand or leaf litter, cardboard walls inclined outward by 15°
^30^	Far-field human speech recognition	Circular MEMs Microphone Array, 7 Microphones in total (Amazon Echo Dot22)	Unknown, but expected to be low	On Device	Noisy indoor environments

An non obvious observation that can be made from Table 2 is that the immediate recording environment influences the sound level. While Briceño *et al*. were able to record the very low-level calling songs of tiny fruit flies using an inexpensive consumer-grade microphone^15^, Goerlitz *et al*. used highly specialised and costly low-noise acoustic measurement equipment to record insect walking sounds^16^. A key difference between these two studies is the acoustic reflectiveness of the surfaces around the sound source. While the Petri dish used by Briceño *et al*. will reflect every sound, resulting in a mechanical amplification of the signal^15^, the insect walking sound recorded on sound-damping substrates in the study presented by Goerlitz *et al*. will attenuate the signals and therefore necessitate higher-quality recording equipment^16^.

As in the previous paragraph, benchmarking anechoic boxes, there is a benefit to extending the benchmark to a broader range of related research fields. Recent advances in human speech recognition have seen intense use of microphone array assemblies to improve classification results in noisy environments or over greater distances^17,18^.

For this reason, applications such as conference call microphones and home-voice assistance usually use four, six or more microphones arranged in a line, a cycle or a sphere. An example is the Amazon Echo Dot device listed in Table 2.

A microphone array is a spatial assembly of multiple microphones. Any simultaneous recording of an acoustic event through multiple microphones with known relative positions contains information regarding the spatial position of the sound source through the phase shift and sound level differences in the sound waves on different channels. This information can be exploited through complex algorithms such as beamforming. These algorithms allow not only the spatial location and temporal tracking of sound sources but also their acoustic separation^19^. If applied to separate signal and noise sources, such algorithms can drastically improve the effective SNR of the recordings and thereby improve classification model performance^20^.

#### Design goals

As with the previous reviews, defining the design goals of the recording setup can be divided into two main parts: recording environment and recording equipment.

**Regarding the recording environment**, the requirements can be further divided into requirements regarding the isolation of the recording environment from external noises and the suppression, or at least control, of reverberations of the target sound within the recording environment.

The review of anechoic boxes built by related projects, as presented in Table 1, shows that the frequency range of the target sound to be recorded within the enclosure has a significant influence on the requirements for sound attenuation provided by the enclosure. While shielding against higher-frequency sounds can be relatively easily achieved, especially by employing double-wall designs, shielding against lower-frequency sounds and even building vibrations requires heavier materials and vibrational isolation solutions, making it increasingly difficult and expensive as the target frequency range decreases.

This situation leads to defining the target frequency range for the insect recordings that shall be enabled by the anechoic box to be built. From a first-principles physics perspective, most insect sounds fundamentally differ from most sounds used to recognise birds, mammals or humans. While the latter possess dedicated vocal organs that allow them to generate sound by blowing air along an organ, which is thereby stimulated to vibrate, most insects of interest in this study do not possess such vocal organs^21^. Furthermore, most of the insects of interest in pest management contexts do not actively communicate using acoustic signals. Instead, the sounds that need to be utilised to identify these insects are an involuntary byproduct of the insects moving their body parts, mostly their wings.

Therefore, the upper limit for the base frequency of most insect sounds is the upper limit of muscle speed. This neuromuscular limit of muscle contraction was found to be approximately 1000 Hz in the fast asynchronous muscle systems that many insects use to fly^22^. Common wing-beat frequencies for the insects of interest in this project range from 77 Hz for the lady beetle *Coccinella septempunctata*^23^ to 180 Hz for small flies such as the greenhouse white fly *Trialeurodes vaporariorum*^24^. Somewhat counterintuitively, this finding means that most insect sound signals are of a much lower frequency than those of much larger and heavier creatures^21^. With regards ot the setup of an appropriate recording environment, these considerations mean the target frequency range is even below 100 Hz.

Considering this very low, and therefore challenging, target frequency range and the results of different designs presented in Table 1, it must be concluded that a recording environment with perfect noise shielding cannot be built with the limited resources of this study. Therefore, monitoring the outside noise level and intervening if it rises above a level that penetrates through the box walls, as proposed by Mankin *et al*., seems wise^12^.

Regarding the attenuation of reverberations within the recording environment, the echo of the surrounding environment of any recorded sound source significantly influences its perception. Considering a real-world application scenario, it can be assumed that the sound emissions from small insects recorded in a greenhouse environment will be very low in intensity. These low-level sounds will only travel a very limited range before degrading below the sound level detectable in the recordings. The objects within this limited range that influence the signal through reverberations on their surfaces are mainly leaves and plants. These objects mostly cause mixed and multidirectional sound reflections, which will lead to an attenuating effect on the signal^25^. Therefore, with good approximation, recordings made under near-free field conditions will best resemble the insect signals in a greenhouse. The term free-field conditions, in acoustics, refers to a recording environment that causes almost no reverberation.

However, insects need to be kept inside some type of container. Preliminary tests with acoustic recordings of insects placed in different small containers, such as Petri dishes, showed a significant influence of these immediate artificial surroundings on the recorded sound. Not only did flight sounds appear to be much louder due to the reflection of sound waves from the rigid walls around the sound source, but otherwise inaudible sounds, such as the footsteps of the insects on the Petri dish surface, were amplified by the surroundings.

With the goal of this work being to create a laboratory dataset enabling the acoustic recognition of insects in greenhouse environments, these heavy artificial influences on insect sounds need to be mitigated. Regarding the recognition task in the greenhouse, sounds that stem from the insect interacting with the laboratory test setup are useless to the dataset, as they will not be encountered under application conditions. Furthermore, the resonance frequencies of insect containers under laboratory recording conditions amplify and dampen different frequencies of the recorded insect sounds, thereby altering the acoustic frequency fingerprint. For this reason, such recordings are unsuitable for building a recognition system that can operate under real-world conditions.

In conclusion, adding obvious project constraints, the following bullet points summarise the requirements regarding a recording environment that allows the noise-shielded creation of a high-quality insect sound dataset for acoustic insect detection:

- Shielding against environmental noise at frequencies as low as 50 Hz is maximised.
- The outside noise level is monitored.
- Reverberations inside the recording environment are minimised.
- The influence of the insect enclosure and of all nonnatural objects with which the insects interact on the recorded sound are minimised.
- The direct surroundings of the insects are arranged to resemble the surroundings under real-world greenhouse conditions as closely as possible.
- Movement of the lab assembly is allowed (e.g., by forklift).
- The design allows clearance of building doors.
- A budget of <1500 € is set.
- The build is completed within <3 months.

**Regarding the recording equipment**, one must consider that the value of the dataset and the number of results that can be generated from it will increase with better hardware. Taken to the extreme, using the best hardware available for this task would allow for more definitive statements about the feasibility of recording such low-level sounds. The same can be said about the data evaluation enabled by such recordings and their usability, e.g., for acoustic classification. Especially in the case of negative results, these results would be of greater impact if they were obtained using the best hardware available. Using the best available hardware eliminates the possibility of better hardware improving the results. In conclusion, the requirements regarding the recording equipment are summarised as follows:

- The edge of today’s recording technology is highlighted by selecting the best-suited recording equipment available.

#### Implementation

**Fig. 1:**
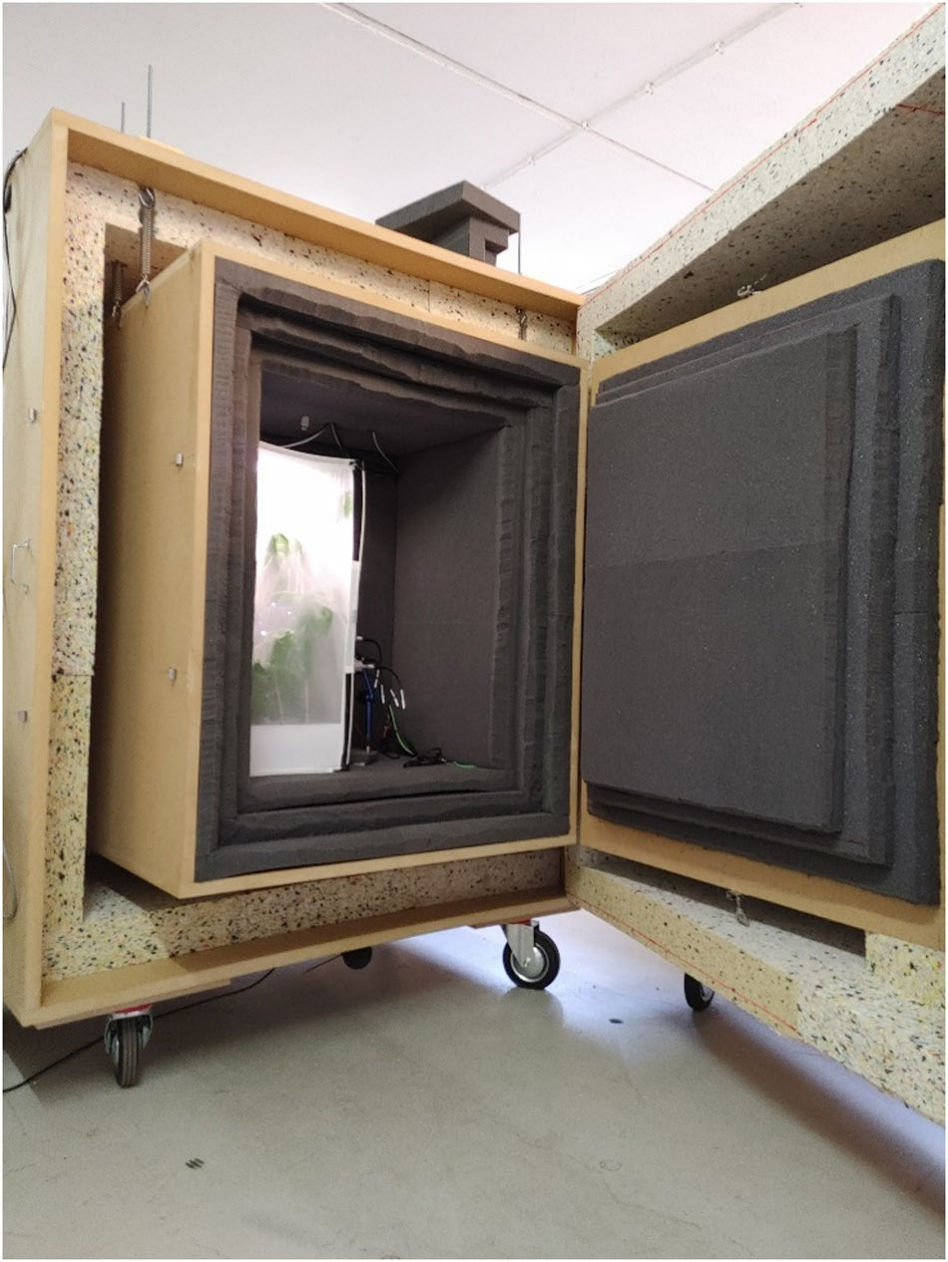
Recording setup for the InsectSound1000 dataset. Double-wall anechoic box containing a microphone array and insect rearing cage.

In this section, the implementation of the recording setup designed for the creation of the InsectSound1000 dataset is presented. The design of the presented setup directly results from the findings and considerations made by reviewing related projects and formulating the design goals, as presented in the previous sections. Figure 1 shows the final recording lab setup.

**The design of an anechoic enclosure** as the basis for an acoustic recording environment was the best way to meet the goals formulated in the Design Goals paragraph. As is evident from the review of related projects in Table 1, such a box will drastically minimise the influence of environmental noise on recordings. Table 3 summarises the properties of the anechoic box built and used to record the InsectSound1000 dataset. The table details the thickness and material density of the different layers.

**Table 3:** Properties of the anechoic box used for the InsectSound1000 recordings.

Source	Layers	Total weight	Special solutions	Achieved sound absorption
Proposed solution	Double-wall: 1. 28 mm MDF wood panel (720 kgm^3^) 2. 100 mm high density composite foam (120 kgm^3^) 3. 100 mm air gap 4. 25 mm MDF wood panel (720 kg/m^3^) 5. 3×50 mm open-cell acoustic foam (30 kgm^3^)	>620 kg	• Inside box suspended from springs inside the outside box • Outside mic to monitor outside noise • Special doors to fit through building doors	Above 50 dB for frequencies above 100 Hz

The first step of the box design involved the calculation of room modes. A web tool (https://trikustik.at/raummoden-rechner/) was used to compare the distribution of axial and tangential room modes for different box dimensions. The inside dimensions of 1170 mm × 960 mm × 750 mm were chosen.

Second, different scenarios of double-wall wooden box designs were evaluated regarding their theoretical sound attenuation potential and resonance frequency distribution. Because of their easy handling, low price and reasonable acoustic properties, Medium-density fibre (MDF) wood panels were chosen as the primary construction material for the double-wall structure. The inside box was constructed from 25 mm panels, while the outside box was constructed from 28 mm panels.

It was decided against using rock or glass wool between walls, even though this is one of the least expensive and most effective ways to improve sound isolation. However, because rock or glass wool is potentially irritating to human skin and respiratory organs, this would make interacting with the box much less pleasant. Instead, a 100 mm thick layer of high-density composite foam was used to absorb reverberating sound between the walls and increase the mass of the outside box walls to absorb low-frequency environmental noise. The air gap from the foam to the inside box was chosen as 100 mm. This gave the double-wall setup an first interwall resonance frequency above 1500 Hz outside the previously identified priority target frequency range.

The inner box was lined with three 50 mm thick layers of open-cell acoustic foam to absorb reverberations and shield against high-frequency environmental noise. Due to the low sound level of the target sounds and their low frequency, which necessitated the use of very long acoustic wedges, such wedges were found to be impractical and unnecessary. The different foams were attached to the wood panels using spray glue. When gluing together the different layers of the open-cell foam inside the inner box, a minimal amount of glue was used in patterns alternating every layer to prevent the sealing of large surface areas of the foam.

The inner box was then suspended by springs from the top of a second larger box, following the example given by^13^. The springs had a spring rate of 4.634 Nmm^−1^, resulting in a normal mode of the spring mounted system of approximately 1 Hz.

While the inside box had a typically designed door, the outside box was split into a door and a box so that the remaining parts were narrow enough to fit through the doors of our building. To minimise the acoustic leakage of the doors, the inside box door was built with a labyrinth-style seal, resulting in a three-layer pyramid of foam on the door panel. The outside box door was sealed by letting the foam overlap 120 mm from the door into the box body, which also resulted in a labyrinth-style seal. The main part of the outside box was placed on four heavy wait transport rolls constructed from rubber to allow relatively easy movement of the box. The outside box door was also placed on two rolls that support the door. Cables were routed through the tops of the boxes via holes that were filled with foam after the cables were routed through. The assembled box has a mass of 620 kg, not including the rolls, springs and bolts used for assembly.

**Microphone choice and array design** was challenging, as many insect sounds tend to be of extremely low volume. The challenge regarding the choice of recording equipment is to maximise microphone sensitivity while minimising self-noise in the measurement chain. Doing so will maximise the SNR. Many insect sounds will be so quiet that they will compete against the self-noise of the system. Standard measurement microphones typically offer a sensitivity of 50 mV Pa^−1^ and a self-noise of 15 dB(A). Special low noise measurement microphones can reach sensitivities of more than 1000 mV Pa^−1^ and self-noise levels below 7 dB(A).

These two parameters influence the recordings of the insects. The microphone sensitivity determines the radius within which a sound of a given amplitude can be captured. The self-noise determines the radius around the microphone within which such a sound can be recognised from the recorded signal. There are many ways to reduce and filter noise in a digital recording. These methods improve this second threshold in postprocessing, while the first threshold cannot be influenced. A signal that is not at all picked up by the microphone cannot be improved. For this reason, the microphone sensitivity was prioritised over its noise level in the selection process.

Considering the background on microphone array signal processing given in the overview of the methods used in related research, it must be concluded that using only one single microphone can never be considered the optimum solution. Furthermore, instead of using a single microphone, recording the dataset with a microphone array would allow the dataset to not only capture the time and frequency aspects of the different insect sounds but also to record information about insect movements, adding to its value.

Based on these findings, special low-noise microphones made by Brüel & Kjaer, called type 4955, were chosen, which, to our knowledge, offered the highest sensitivity on the market as of the time of selection. Because of the previously described benefits of using an array of microphones over a single microphone, it was chosen not only to use a single microphone but also to assemble four of them to form an array. The four microphones used to record the InsectSound1000 dataset offered a sensitivity of more than 1300 mVPa^−1^ at a self-noise level of 6.5 dB(A).

The four microphones were connected to a Brüel & Kjaer Nexus 2690 conditioning amplifier, which supplies the microphones with the conditioning voltage for the microphone capsules and the supply voltage for the microphone preamps. The Nexus unit also offers an analogue low- and high-pass filter option, as well as an integrated analogue amplifier. For the digital analogue conversion, an A-D converter made by Roga Instruments, called DAQ4, was employed. This unit was connected to a standard office notebook running the software DasyLab to control the DAQ4 and manage the recording process. Table 4 summarises the recording setup.

**Table 4:** Overview of the recording hardware employed to record the InsectSound1000 dataset.

Source	Target Species and Sound	Microphone Type	Microphone Sensitivity	Data Acquisition	Recording Environment
Proposed solution	Greenhouse pest and beneficial insects	1/2” low-noise measurement microphone	>a1300 mV Pa^−1^	Analog pre-filtering, analog pre-amplification, 32 A-D-converter	Insect rearing cage with two tomato plants inside Anechoic Box

**Fig. 2:**
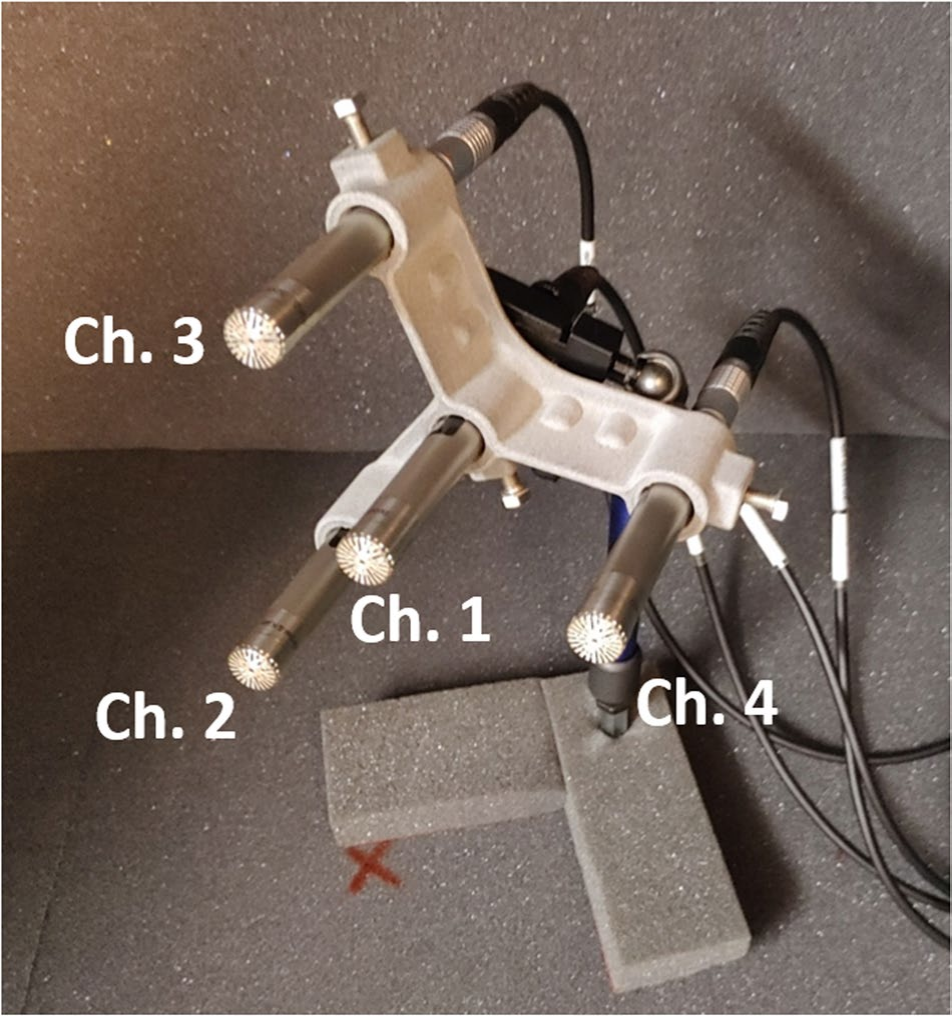
A custom-made microphone array fixture was used to assemble four low-noise measurement microphones, pictured here inside the anechoic box. The channel numbers are indicated in the picture.

The four microphones were assembled to form an array using a self-designed, 3D-printed fixture constructed from PA-12, as displayed in Fig. 2. As the distance of the microphones influences the signal processing possibilities through beamforming algorithms, the dimensions and shape of the array must be carefully considered. The four microphones can sensibly be arranged in either a square shape or a star-like shape, with one microphone in the centre and the three other microphones within a constant radius around the centre. The latter design was chosen because it is easier to point such a star-shaped array at the object under test than a square array.

Regarding the array dimensions, larger distances between the microphones will give better beamforming results for lower-frequency sounds. Increasing the distance between the microphones will maximise the measurable phase shift in long low-frequency sound waves, on which beamforming algorithms rely.

As previously discussed, most insect sounds are of relatively low frequency. Furthermore, for practical applications, it is found that the majority of environmental noise is also composed of low frequencies. Our everyday noise environment can be described as 1/f-noise or pink noise. Low-frequency sounds travel further in the open environment than high-frequency sounds, making it more likely that a low-frequency sound originates within the audible distance than a high-frequency sound^25^.

These two arguments suggest that the distance between microphones should be maximised. On the other hand, this suggestion is contradicted by the fact that insect sounds are not only low in frequency but also extremely low in intensity and are often not even audible to the human ear. As the sound pressure level decays reciprocally proportional to the distance to the sound source, the distance between two microphones can quickly reach a dimension where a low-level sound can only be picked up by one of the microphones. Notably, the precondition for using any array algorithm is that the signal must be present in more than one of the recorded channels.

Hence, it is difficult to determine an optimal solution for this conflict of objectives, especially without a considerable amount of data about the use cases encountered in practice. Therefore, an educated guess had to be made. According to Tager^26^, a basic delay and sum beamforming algorithm will yield good results for a range of

$$0.25 < \frac{d}{\lambda } < 0.5$$

where *d* is the interelement spacing of a linear array and *λ* is the wavelength of the sound signal. Based on these calculations, the three outside microphones of the measurement microphone array were placed on a radius of 55 mm from the fourth microphone in the centre. This positioning results in a distance of 95.26 mm between each of the three microphones on this radius. This distance should give the device a beamforming frequency range of 900 Hz to 3118 Hz according to these conservative estimates. While this frequency range is above the previously derived target frequency range for insect flight sound base frequencies, it will contain most of the harmonics of these insect sounds.

To monitor environmental sounds outside the anechoic box during the recordings, a fifth measurement microphone was placed underneath the box. For this task, a low noise measurement microphone (Microtech Gefell, MMS 2014) available from preliminary experiments was employed. Connected to a Plug.n.DAQ unit made by Roga Instruments, this outside channel could be simultaneously recorded to the four microphones inside the box using DasyLab software.

**The choice of insect enclosure and the design of a recording environment** needed to be carefully considered. In an effort to characterise the influence of different insect containers on laboratory sound recordings, an artificial sound source was created from 6 in-ear headphone speakers. The headphones were placed in a small lump of putty in such a way that one speaker was facing every room direction. The design was intended to mimic the setup of room acoustic speaker cubes used to measure and test room reverberations in architectural applications.

This artificial sound source was placed in different insect containers while playing a sine sweep sound over these speakers. The resulting sounds were recorded using a measurement microphone. The insect containers that were tested included Petri dishes of different sizes, take-out-soup dishes made from styrofoam, insect-rearing bags made from mesh material and different insect-rearing cages. For a more realistic test, the same containers were also recorded using real insects as sound sources. The results were too incoherent to justify a publication in this context. However, generally, the obvious relation that smaller containers with more closed surface areas would more strongly alter the signal was evident in the recordings.

**Fig. 3:**
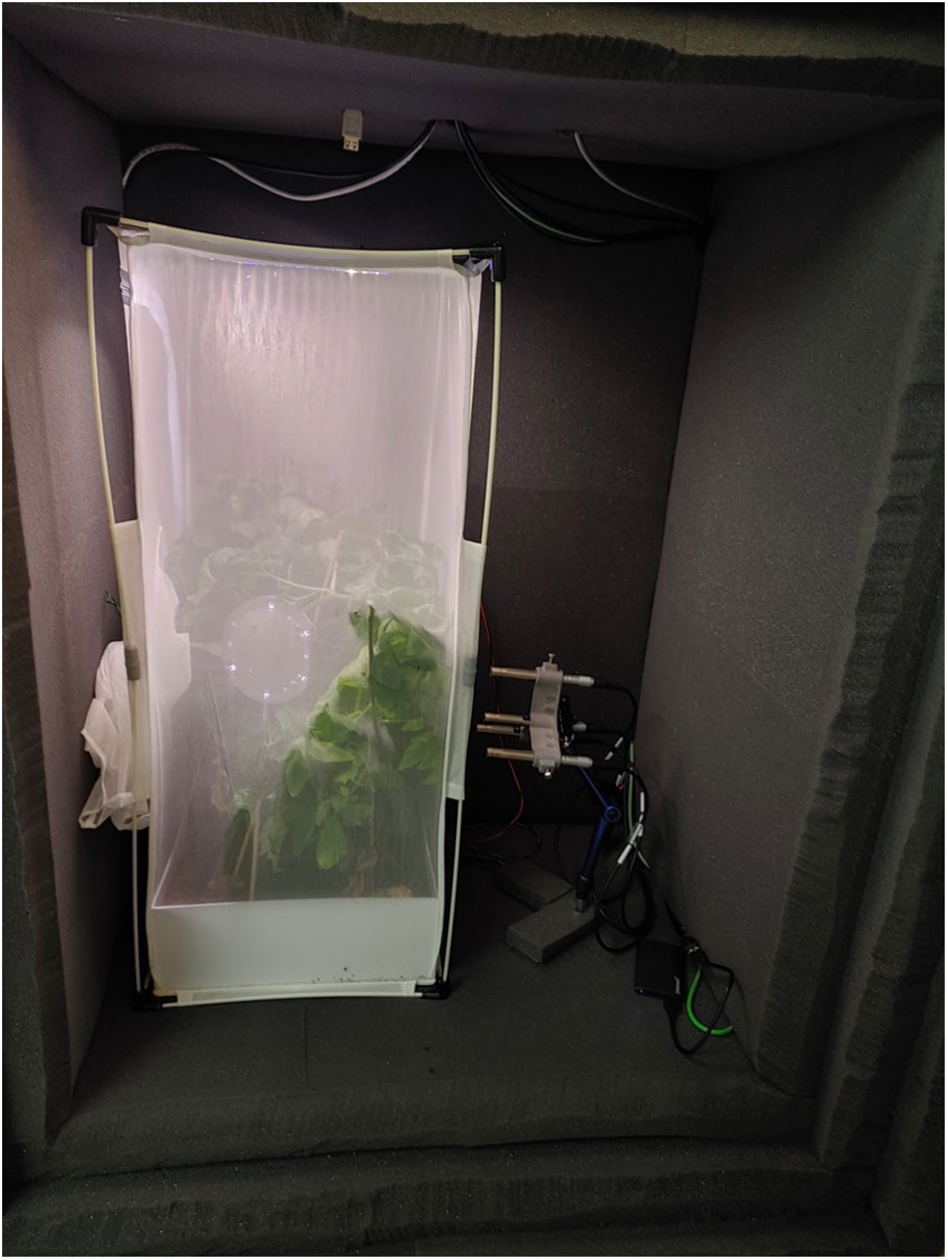
View of the recording setup for the InsectSound1000 dataset inside the anechoic box. An illuminated insect-rearing cage containing a square plant pot with two tomato plants, a low-budget microphone array and one selected species of insect was placed inside the box to the left.Next to it is the high-quality microphone array, which consists of 4 low-noise measurement microphones.

Figure 3 shows the final recording setup inside the anechoic box. In conclusion, an insect-rearing cage constructed from mesh material and sized as large as possible was used to fit the anechoic box. The cages had a size of 32.5 cm×32.5 cm×77 cm. The mesh walls minimise reverberations and footstep sounds. Furthermore, the larger size places the walls farther away from the sound sources. Given the low level of insect sounds, this placement should reduce reverberation from walls because the amplitude of the sound reaching the wall from a distance will, in many cases, already be very low. The downside of using a larger container for insect recordings is that with the very low sound level of many insects, this reduces the likelihood of an insect being close enough to the microphones to allow their signal to be picked up. This disadvantage had to be balanced by increasing the number of insects in the container and the recording time.

Following the previously described argument that everything artificial within the laboratory recording setup will likely negatively alter insect sounds, the goal was to keep the surroundings of the insects within the rearing cage as close to the greenhouse environment as possible. Therefore, a custom square plant pot was made from PVC plates to exactly fit the dimensions of the cage. Within each plant pot, two small tomato plants were planted in diagonal corners. Having plants and soil inside the cage should allow the recording of sounds that stem from insects’ interactions with plants and reduce the number of sounds that stem from insects interacting with the cage, e.g., by walking on the mesh. After problems with fungas gnat infections in the potting soil, the soil used to plant the tomato plants was disinfected by microwaving it for 200 s at 900 W. Clay pellets were placed at the bottom of the plant pot to prevent dammed-up water. In total, six identical cages were set up as described and inoculated with different pest and beneficial insects.

The microphone array was mounted to a fixture for easy positioning. Using the fixture, the microphone array was placed approximately 30 cm above the box and insect cage floor. The microphones were placed directly facing the cage, very close to but not touching the cage mesh.

Two types of LED stripes provided the light inside the box to stimulate natural daytime behaviour in insects. The strips were glued onto a small aluminium plate as a heat sink and placed on top of the cage. The LED strips produced visible light (CRI = 95%) and UV light. The light setup was slightly changed for recordings of *T. vaporariorum*. As the LED lights strongly attracted these insects, the LED lights were positioned on the sidewall of the rearing cage at the height of the microphones to guide these insects closer to the microphones. Due to their very low-level flight sounds, the original recording setup with the lights placed on top of the cage turned out to be unsuccessful, as insects flying around the lights placed on top of the cage were too far from the microphones to be recorded.

Apart from the measurement microphones used to record the InsectSound1000 dataset, a second low-budget microphone array was present inside the anechoic box during each recording. In the context of the IPMaide project, the recording capabilities of a Seeed Respeaker Core v2.0 MEMS microphone array were tested alongside the high-quality microphone array described at length in this publication. The MEMS microphone array was housed in a custom-made 3D printed housing made from PA-12, which included an aluminium cooling heat sink and fine mesh inserts to protect the microphones. This device was placed inside the rearing cage during every recording session. Unfortunately, this device created a low-level but constant sound signal with two distinct peaks at approximately 10.7 kHz.

Because these sounds are outside the target frequency range, are constant and are present in every single recording, they should not influence the value of the InsectSound1000 dataset. This is neither for the distinction of different insects under laboratory conditions nor for the transferability of models trained on InsectSound1000 to real-world applications. Because the InsectSound1000 sample dataset is downsampled to a sample rate of 16 kHz, from the originally recorded 48 kHz, the sounds of the Respeaker Core v2.0 array are not audible in any of the InsectSound1000 samples (see Recording Process section). They are, however, audible in the original raw recordings and would appear in any version of the dataset processed with a sample rate greater than 20 kHz. As these sounds represent a deviation from real-world conditions, this could have a negative, although minimal, effect on the transferability of models trained on such 20 kHz-or-above-datasets to real-world applications.

### Recording process

Apart from microphone choice, the measurement equipment used to power the microphones and process the analogue signals has a large influence on recording quality. Each item in the measurement chain was carefully chosen to ensure that the recordings made for the InsectSound1000 dataset could exploit the full potential of the high-quality microphones that were selected. Preliminary experiments were conducted to determine the optimal settings for each component. The following is a description of the resulting items and their settings. Furthermore, the recording schedule and insect handling are explained.

#### Recording hardware settings

The microphones were powered by a Brüel & Kjaer Nexus 2690 conditioning amplifier. This unit also offers an analogue low- and high-pass filter option, as well as an integrated analogue amplifier. The Nexus high-pass filter was set to 20 Hz, and the low-pass filter was set to 10 kHz. To minimise the noise introduced by the Nexus amplifier, one must employ a special technique. From the data sheet supplied with the Nexus, it is evident that the self-noise introduced by the amplifier is minimised at gain levels that are a whole-number multiple of 10 dB. As the Nexus, by default, is set up to equalise the sensitivity of every channel by adjusting every channel’s gain to pull all microphones onto the same output sensitivity, one has to bypass this function to be able to explicitly set the gain level for every channel. This effect can be achieved by setting the correction factor on the Nexus for each microphone to the inverse of its microphone’s sensitivity. By setting up the Nexus this way and choosing an output level of 10 VPa^−1^ on the Nexus, the signal of every channel was amplified by 20 dB while introducing the minimal possible amount of amplifier noise into the signal.

The analogue low- and high-pass filtered and amplified signal was then fed to an A-D-converter manufactured by Roga Instruments called DAQ4. The gain function on DAQ4 was disabled, as the preliminary test showed that amplification using DAQ4 would result in more noise than amplification at the same level on the Nexus. Furthermore, the DAQ4 was set to AC-coupled mode, and a digital high-pass filter with 0.3 Hz was activated. All recordings were digitalised at a sample rate of 48 kHz. The DAQ4 was set up and recorded using the software DasyLab 2022 installed on a standard office notebook. The recordings were saved as single-precision (32-bit, floating-point) TDMS files, each of which were 14:13 min long. The TDMS-file format is a file format for measurement data introduced by National Instruments, who offers a Python library that allows for easy further processing and file-format conversion of these files.

#### Recording schedule and insect handling

The recordings were collected overnight to minimise the influence of noise from people working in the building. The recording period started in the afternoon and ended in the morning. The light inside the box was controlled via a time switch and set to turn off at 3:00 in the morning. To account for the night recordings, the light schedule in the climate chambers where the insect cages were kept when not recorded was delayed by three hours. This delay was intended to shift the circadian rhythm of the mostly diurnal insects towards later in the day to increase insect activity during the night recordings.

For every recording, only one cage containing 2 to 50 insects of one species was placed inside the anechoic box. Larger, louder and more active insects were recorded using only a few individuals, while smaller and less active insects were recorded in greater numbers to ensure that three to four nights of recording would yield roughly the same amount of insect sound events in the recordings. All insects were randomly selected from either their captive breeding cages or by catching them in the wild. The insects were not separated by sex or age. For larger insects with a longer lifespan, the same individuals were usually reused for all different recording nights. Smaller insects were recorded in greater quantities. For these smaller insects, much shorter lifespans meant that the population in the recording cage fluctuated between different recording nights, and counting the number of individuals was sometimes challenging.

### Data processing

The InsectSound1000 dataset was created from the raw, long-form and mostly silent recordings made insight the anechoic box by automatically extracting sound events. The extracted sound events were downsampled and saved in an accessible file format. This process is detailed in the following section.

To simplify processing with simple sound sample classification models, all sound samples in the dataset need to be of the same length. As the length, and therefore the data size, of the extracted samples greatly limits the complexity of model architectures to be explored because of memory constraints, a rather short sample length of 2500 ms was chosen for the samples in the InsectSound1000 dataset.

To further limit the sample data size and thereby accelerate every form of processing the dataset, the samples were downsampled from the initially recorded 48 kHz signals to 16 kHz. As discussed in the design goals section, most insect sounds are expected to be of very low frequency. Therefore, downsampling to 16 kHz, which should still adequately resolve signals up to 8 kHz according to the Nyquist-Shannon theorem, is expected to have minimal impact on the information content of the sound dataset. The segmentation process, based on the idea of activity detection on a prefiltered signal employed by the sample extraction program, was loosely based on the steps described in^27^. The sound sample extraction program implements the following steps:

1. One of the 14:13 min long TDMS files is loaded.
2. The signal is downsampled from 48 kHz to 16 kHz.
3. The loudest channel of the four channels recorded is selected by summing the squared values of every channel and selecting the channel with the maximum squared signal sum. By reducing the signal from four channels to one channel, the computational expense of the following steps can be drastically reduced.
4. Prefiltering the signal by passing it through a 4^*th*^ order Butterworth low-pass filter at 1500 Hz and a 30^*th*^ order Butterworth high-pass filter at 180 Hz. Prefiltering minimises the influence of environmental sounds that did leak through the anechoic enclosure on the following steps of processing.
5. The signal is windowed, and the signal energy within each window is estimated by calculating the signal energy for every window as the sum of the squared signal values. A window size of 3279 values, hopped by 1024 values, was employed for this step.
6. Activity detection by thresholding the window energies. The threshold for window energy was set to 1.6 times the mean of all the window energies found in the 14:13 min recording. Parts of the signal that exceed this energy threshold are marked as “activities”.
7. Nonoverlapping, equal-length sound samples containing one or multiple phases of “activity” are extracted. For this segmentation process, a cascade of if-else cases is used to divide the signal into nonoverlapping samples of 2500 ms in length based on the previously calculated activity markers. Segments shorter than 1 s without any previous or following activity segments within a 2500 ms range are discarded as noise.
8. The extracted sound samples are saved as WAVE files. The final sound sample file, stored on a disk, contains the original, unfiltered, four-channel signals of the extracted segments at a sampling rate of 16 kHz.

Notably, the sounds recorded by the fifth microphone mounted outside the box to monitor the environmental noise level during recordings have not been involved in the sample extraction process to date. Instead, an aggressive, very high-order, high-pass filter was applied before activity detection. As only very low-frequency outside noises are expected to pass through the anechoic box walls, this should prevent most critical noise events from triggering sample extraction. However, given the presented sample extraction program, it is possible for any of the samples extracted to contain outside noise along with an insect sound that triggered sample extraction.

However, the effect of these outside noises on the usability of the InsectSound1000 dataset for training deep learning models and their application is believed to be minimal. Each insect species was recorded multiple nights to reduce the likelihood of similar background sounds occurring in every recording of one species, which would create bias in the dataset. Therefore, the noise sounds that are randomly extracted with insect sounds should be randomly distributed among all classes and, therefore, should not affect any model training. However, future work could exploit the use of outside microphone data to completely prevent any outside noise from entering the extracted insect sound samples.

## Data Records

The InsectSound1000 dataset is stored on OpenAgrar^28^, a collective, open-access repository of research institutions affiliated with Germany’s Federal Ministry of Food and Agriculture (BMEL). OpenAgrar is operated by, among others, the German Federal Research Centre for Cultivated Plants (JKI), where the sounds were recorded in the context of a research project called IPMaide.

Unfortunately, due to the immense amount of raw audio data, the InsectSound1000 can only contain extracted sound samples. Readers interested in the long-form, raw audio recordings, amounting to roughly 6 TB of data, will be encouraged to contact the corresponding author. We are happy to provide individual solutions for accessing the raw data via, e.g., shipment of hard drives. In combination with the published sound sample extraction code (see section Code Availability), every data processing step, from raw audio data to sound samples, can be reproduced and modified.

In total, 1079 h of sound recordings of 12 different insect species were made using the process described in the Methods section throughout the 72 recording nights. Using the sample extraction program detailed in the data processing section, these recordings were processed to generate the InsectSound1000 dataset. Overall, 165982 sound samples were extracted from the recordings.

The choice of insects was based on the following considerations. The purpose of the original study was to build a system applicable in the context of protected horticulture. Therefore, the insects chosen are likely to be found in a European greenhouse. Furthermore, the goal was to portray a range of different sound levels. Selecting insects of different sound levels should allow for a more detailed analysis of detection model performances and their limitations. The dataset should also highlight the influence of different degrees of kinship. If it contains closely as well as distant related species, the dataset will enable more nuanced statements regarding the limits of acoustic distinguishability. Therefore, not only *Halyomorpha halys* and *Nezara viridula* were recorded but also species that can easyly be confusion visually with these insects, such as *Rhaphigaster nebulosa* and *Palomena prasina*.

Table 5 lists the names, orders, families and species of the 12 different insects contained in the InsectSound1000 dataset. The approximate number of individual insects that were placed inside the cage during the recordings is listed. The number of hours recorded and the number of samples that could be extracted from the recordings for each of the 12 insect species are also listed. Furthermore, a column shows the average number of samples that could be extracted per recording hour for each insect, calculated from the previous two columns.

**Table 5:** Numeric overview of the InsectSound1000 dataset, detailing the different insect species, their recording time and the number of samples that could be extracted from the recordings.

Insect Order	Insect Family	Insect Species	No. of insects inside the cage during recording	Recording time [h]	No. of samples in the dataset	Average No. of samples extracted per recording hour	Mean sample SPL in dataset [dB]
Hymenoptera	Apidae	Bombus terrestris	5	48	18291	381	25.9
Diptera	Syrphidae	Episyrphus balteatus	5–15	99	16868	170	18.5
Diptera	Cecidomyiidae	Aphidoletes aphidimyza	20–30	84	14065	167	10.7
Diptera	Sciaridae	Bradysia difformis	20–30	47	11394	242	9.3
Heteroptera	Pentatomidae	Rhaphigaster nebulos	3	94	18445	197	11.8
Heteroptera	Pentatomidae	Palomena prasina	6	158	27340	173	11.6
Heteroptera	Pentatomidae	Halyomorpha halys	4–6	74	19671	265	11
Heteroptera	Pentatomidae	Nezara viridula	4	105	20323	194	10.4
Lepidoptera	Gelechiidae	Tuta absoluta	30	126	633	5	13.4
Coleoptera	Coccinellidae	Coccinella septempunctata	10	68	14682	217	12
Hemiptera	Aleyrodidae	Trialeurodes vaporariorum	50	95	1062	11	9.7
Hemiptera	Aphididae	Myzus persicae	30	81	3208	40	9.6

**Fig. 4:**
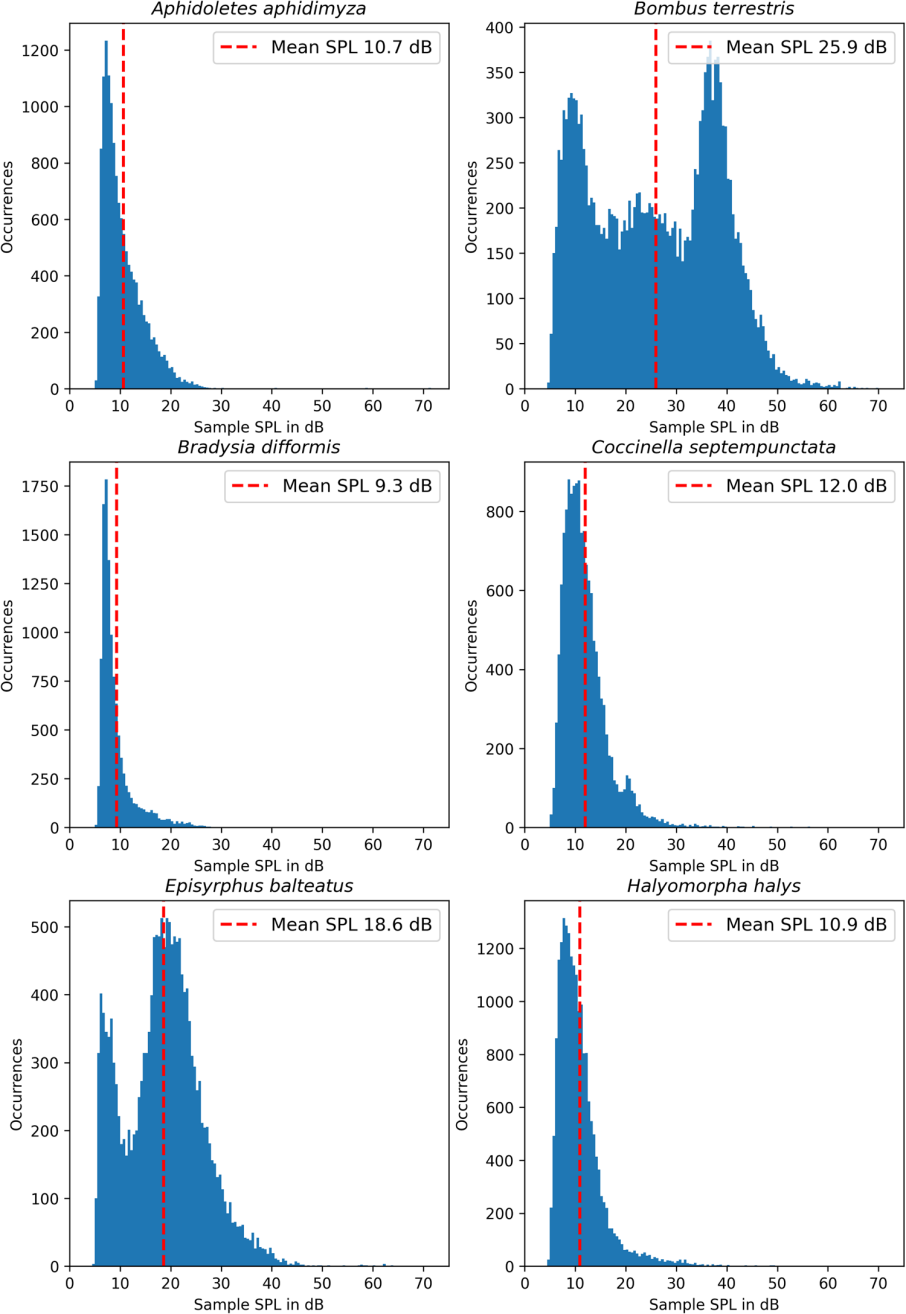
Histograms of the different SPL-values calculated for every sample for the different classes in the InsectSound1000 dataset.

**Fig. 5:**
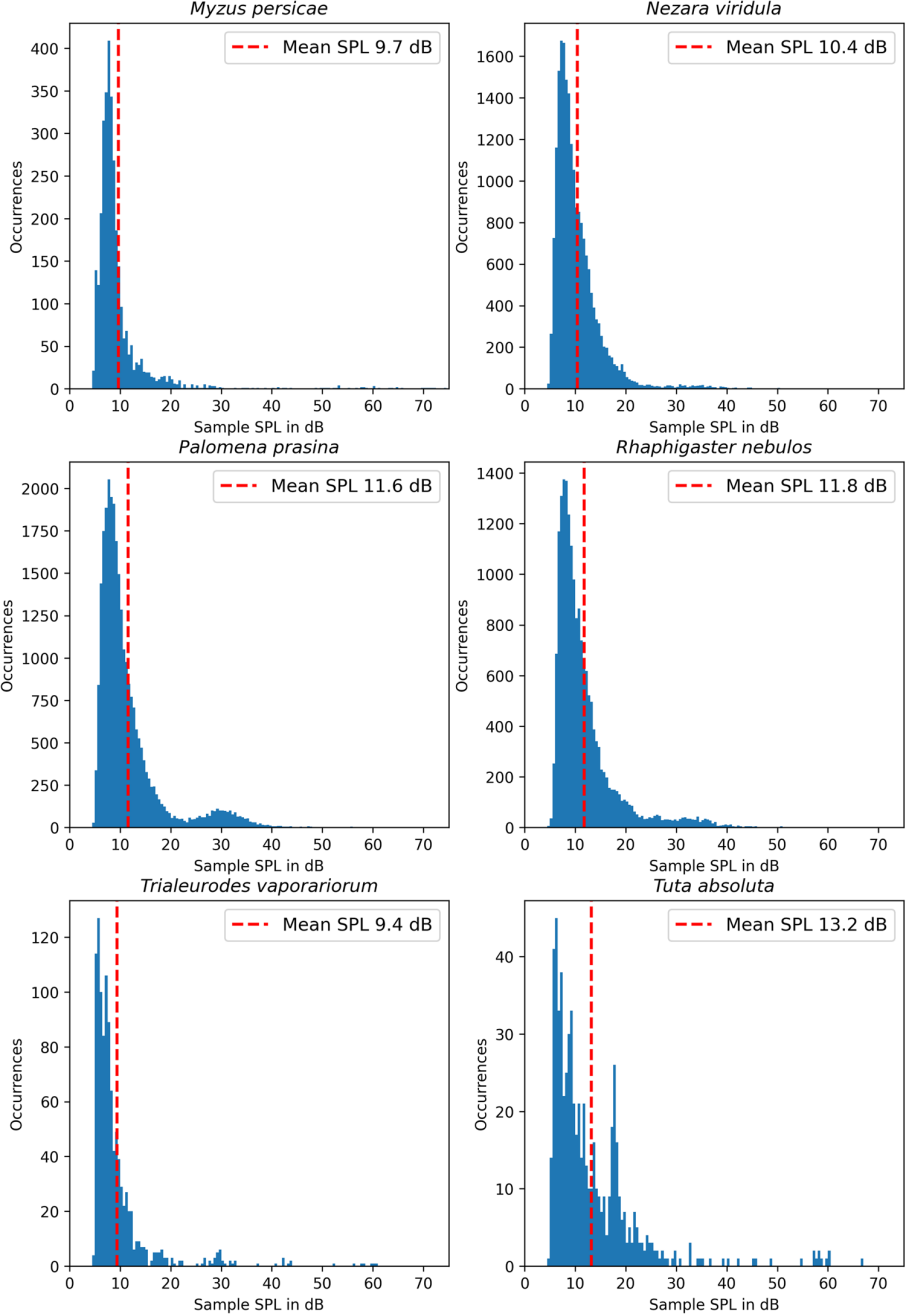
Continuation of Fig. 4.

The last column lists the mean SPL of all samples in the dataset for each insect species. This value is calculated by calculating the mean SPL of every 2500 ms long sound sample in the dataset after applying a 50 Hz, 4^*th*^ order, Butterworth high-pass filter and offset correction. The means of these values are then reported in the table. Figures 4, 5 show plots further detailing the distribution of sound samples over the relevant SPL range for every insect species.

Of the selected insects, four are beneficial insects used in horticulture for pest management (*Aphidoletes aphidimyza, C. septempunctata, Episyrphus balteatus*) or pollination (*Bombus terrestris*). As these insects are deliberately released in greenhouses for their benefit, they are commercially reared and purchased from a local beneficial insect supplier. The remaining insects used for the recordings were considered pest insects and were either captured in nature as adults (*P. prasina, R. nebulos*) or taken from captive breedings (*Bradysia difformis, H. halys, Myzus persicae, N. viridula, T. vaporariorum, Tuta absoluta*).

Table 5 clearly shows that the amount of extractable acoustic activity per recording hour greatly varies among insects. This seems to depend on two factors. The first factor is the overall sound level of the insect. Loud insects, such as *B. terrestris*, could be successfully recorded independent of their position within the rearing cage; quieter insects, such as *A. aphidimyza*, only produce sounds of such low volume that even with the high-quality measurement equipment used, they will very likely only be audible in a very limited range directly around the microphones.

The second factor influencing the acoustic activity detected was overall insect activity and willingness to move. An evaluation of the recordings each day revealed that this willingness to move varied between species of insects but also between different days. Typically, a batch of newly emerged insects was more active than insects near the end of their lives.

### Sound sample properties

InsectSound1000 consists of a single zip folder containing 165982 files amounting to 95.1 GB. Each file has the following properties:

- Length: 2500 ms
- Size: 625 kB
- Channels: 4
- Resolution: 32 bit
- Sampling rate: 16 kHz
- Format: uncompressed WAVE-file
- Filename consisting of:
  - Recording date
  - Full scientific insect species name

The recording equipment used allows the recording of sounds even below the human hearing threshold. An example of this is the very quiet flight sounds of *A. aphidimyza* that are clearly audible in the recordings but inaudible for bare ears. Furthermore, through the use of this highly specialised equipment, the sounds recorded are not limited to the flight sounds most notable to humans. Listening to the recordings, there appear to be many sounds that stem from insect movements other than flight. These sounds are quieter and can be described as rattling or scratching noises. Even though the equipment used allows the capture of remarkably quiet sounds, this setup introduces a considerable amount of amplifier noise into the recordings.

## Technical Validation

This section presents an experiment that was conducted to test and characterise the experimental setup and equipment used to record the InsectSound1000 dataset. The experiment quantifies the noise absorption performance of the designed anechoic enclosure. As this box is self-built and the result of the previously described design process (see the sections on design goals and implementation), there is no other way to evaluate its noise absorption performance than to test it. Analysing the noise absorption performance of the box is also the best way to describe the type of nontarget background noise present in the InsectSound1000 dataset. The reader interested in the detailed characteristics of the Brüel & Kjaer type 4955 microphones used to record the InsectSound1000 dataset shall be referred to the official Brüel & Kjaer data sheet (https://www.bksv.com/en/transducers/acoustic/microphones/microphone-set/4955).

### Method of experimental assessment of noise reduction through the anechoic box

In an attempt to quantify the noise isolation properties of the box build, comparative measurements were made. A speaker and matching amplifier were placed roughly 2.5 m in front of the anechoic box. Inside the box, the measurement microphone array, described in the Implementation section, was positioned facing the door opening. The microphones were connected to the Nexus unit, which was in turn connected to a Roga Instruments DAQ4, as described in the Recording Process section. All devices were set up as outlined in the Recording Process section, apart from the following exceptions:

- The high pass filter on the Nexus was set to the lowest possible setting: 0.1 Hz
- The low pass filter on the Nexus was set to 30 kHz.
- The output level of the Nexus was set to 31.6 VPa^−1^. This resulted in an amplification of 30 dB for every channel.

Measurements were made by playing a WAVE sound file over the speaker in an endless loop. The sound file consisted of a 20 s-long sine sweep sound from 20 Hz to 20 kHz followed by 2 s of silence. The speaker amplitude was chosen to be as loud as possible without clipping the microphone signals with the given setup. Even though the speakers exhibit a highly nonlinear frequency response and cannot produce the same sound level for every sound frequency contained in the sweep sound signal, the sound absorption properties of the box should become apparent by comparing different recording scenarios.

The baseline measurement was made with both box doors open wide. Next, the inner box door was closed, and the second measurement was taken. The outer box door was also closed, and the third measurement was taken. Each measurement was started at a random time while the endless loop of sinus sweep sounds played over the speaker. The test was terminated when a minimum of three full sweep sounds had been measured without any unusual external noise occurring during the measurement time window. This process was repeated until three valid measurements could be made for each of the three test scenarios: the box was fully open, the small door was closed, and the box was fully closed. During the comparative measurements, only one person was in the room, quietly sitting in the same spot every time. None of the objects in the room were moved between measurements, nor were any room doors or windows opened during recording. Recordings during which strong external noises occurred (e.g., a truck driving by) were excluded from the evaluation.

The recorded TDMS files were converted to WAVE files using a custom Python program for data evaluation. Using the software Audacity, the WAVE files were inspected to obtain the time stamps of three sweep sounds with minimal environmental noise in every recording.

To allow the spectral comparison of three different recording scenarios, a Python program was used. The program begins by loading the relevant raw TDMS files. Based on the previously noted time stamps, three 21 s-long segments containing sweep sounds are extracted from every recording, resulting in nine sweep sound clips per scenario with four channels each.

The program then calculates the fast Fourier transform (FFT) for each channel of each sweep sound recording. The calculation results for the three scenarios were averaged for the final comparison. To improve the visual representation of the resulting frequency spectrum plots, moving average smoothing with a window size of 51 data points was applied before plotting the results.

### Results of experimental assessment of noise reduction through the anechoic box

**Fig. 6:**
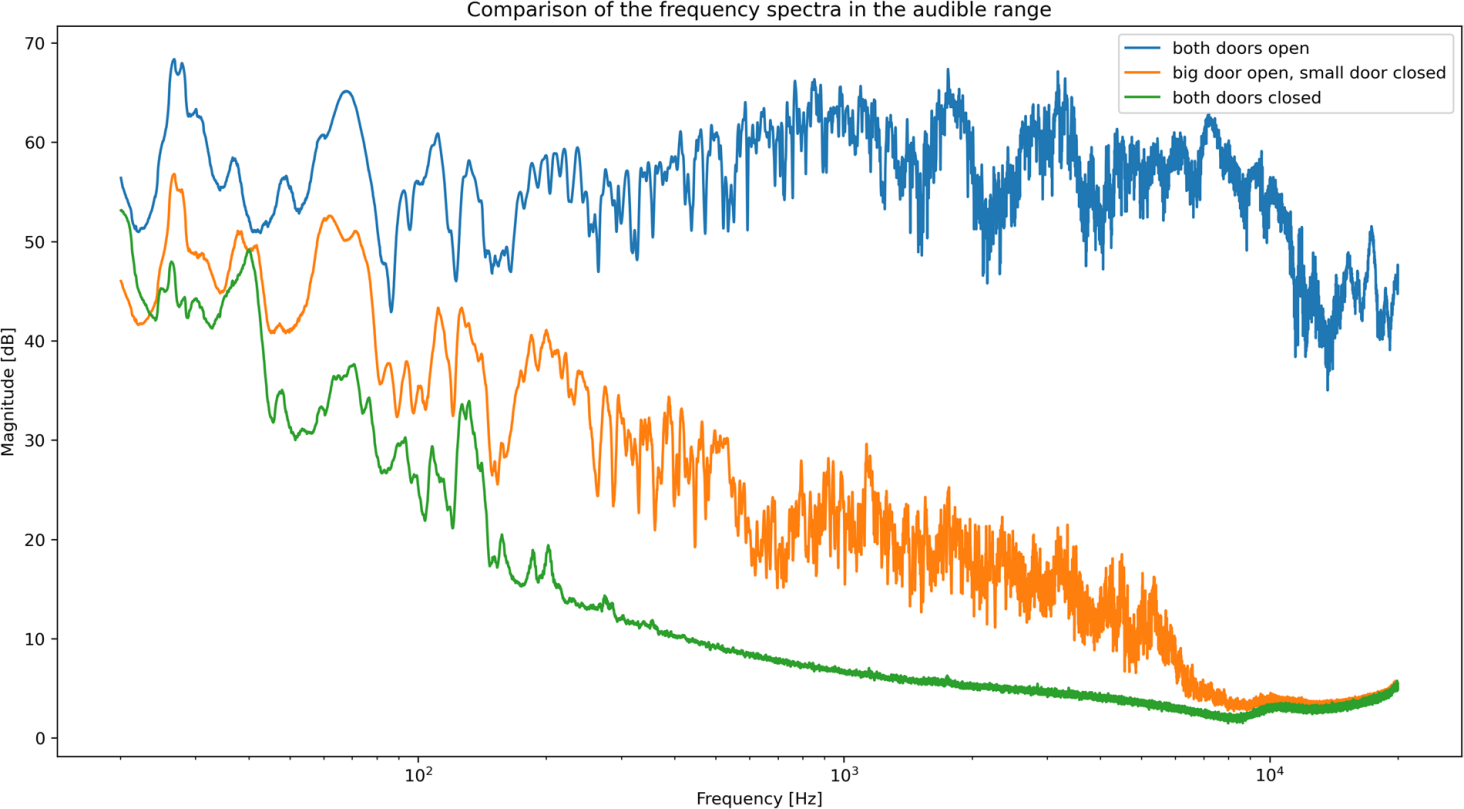
Sound absorption test results. The graphs display the spectrum of the signals recorded by the microphones inside the box with both box doors open (blue), with just the insight box door closed (orange) and with the anechoic box fully closed (green) when a sine sweep sound from 20 Hz to 20 kHz was played outside the box from a speaker.

Figure 6 shows the results of evaluating the sound absorption properties of the anechoic box built with the experimental setup.

The blue plot, representing the measurement with open doors, shows the baseline measurement of the employed test signal. As both box doors were open, the anechoic box should have had little to no effect on these recordings. The blue graph shows high fluctuations in the magnitude of the different frequencies in the recorded signals. It also shows a slightly decreasing magnitude for higher frequencies above 10000 Hz.

Theoretically, the sign sweep signal should produce a test sound of equal magnitude for every frequency, resulting in a completely horizontal line in the blue plot. However, the speakers and the amplifier used cannot produce the same sound level over the entire tested frequency range. Furthermore, the reverberations from the room walls and the objects inside the room further distorted the test signal. Therefore, this experiment can only show relative sound absorption levels. The sound absorption achieved by the anechoic box that was designed and built can be read from the figure as the difference between the blue line and the orange or green line, respectively.

Comparing the blue and orange plots, which represent the measurements with only the inside box door closed, the orange plot shows a decrease in magnitude for the entire frequency range. From the plot over a log scale on the x-axis, it can be observed that the sound absorption achieved by closing the inside box door (read as the difference between the blue plot and the orange plot) increases roughly exponentially with the frequency. Starting with almost no noise reduction at 20 Hz to 45 Hz, the noise reduction increases with increasing frequency. It reaches a level of approximately 20 dB at approximately 200 Hz before increasing to approximately 50 dB at 9000 Hz. Looking at the smoothness of the graph, it can be observed that the orange plot does show a fluctuating magnitude up to 7000 Hz. At higher frequencies, the calculated spectrum appears smoother.

The noise reduction achieved by fully closing the box (read as the difference between the blue graph and the green graph), again, shows almost no noise reduction for very low frequencies and an exponentially growing noise reduction for increasing frequencies. With increasing frequency, the noise reduction achieved by the fully closed box very quickly reaches more than 45 dB. Above 8000 Hz, no difference in noise reduction can be observed between the closed inner box and the fully closed box, as both graphs slightly pitch up for very high frequencies above 10000 Hz. Starting from roughly 300 Hz upwards, the green plot appears very smooth as the magnitude of neighbouring frequencies fluctuates very little.

### Discussion of the experimental assessment of noise reduction through an anechoic box

As described in the Results section, the observation at first glance at Fig. 6 is that while the blue graph shows a very fluctuating spectrum, the majority of the green graph and the section of the orange graph above 8000 Hz appear to be much smoother. A possible explanation for this is the following: while recording the green plot, with the anechoic box fully closed, almost none of the sounds from the speaker placed outside were loud enough to be recorded by the microphones inside the box. As the other two plots show that the test signal played on the speaker fluctuated greatly, it is likely that the smooth sections of the green and orange plots do not represent a recording of the sound played by the speaker leaking through the box but instead show the noise floor of the employed measurement setup. This would also explain why the measurement with one and both doors closed yielded exactly the same measurement above 8000 Hz.

This finding means that the present experiment very likely underestimates the real sound absorption potential of the anechoic box. Furthermore, this underestimation explains why the sound absorption (read as the difference between the blue graph and the orange or green graph, respectively) seems to decrease for frequencies above 10000 Hz, while the underlying physics of sound absorption suggests increasing sound absorption success with increasing frequencies, as presented in the overview of the methods used in related research.

By comparing the sound absorption spectrum of the box with only the inner door closed to the box with fully closed doors, the positive effect of the double-wall structure can be observed. As was expected from the results of comparable experiments and theory presented in the overview of the methods used in related research, good absorption of high-frequency noises can be achieved by using relatively thin and light absorption materials, while the absorption of lower frequency sounds can only be achieved by using heavy materials and/or double-wall structures.

Overall, the sound absorption spectrum achieved by the presented anechoic box must be considered satisfactory considering the project constraints (budget and time). While the results show that virtually no environmental sounds of high frequency (above 1000 Hz) are likely to make their way through the box walls and into the insect sound recordings, the recording setup remains vulnerable to low-frequency sounds. The proposed anechoic box offers little to no noise shielding for frequencies of 450 Hz or below. This finding is consistent with the test results of other builds with a comparable weight range and design presented in Table 1. Low-frequency sound absorption could only be improved by using heavier materials such as concrete or even by building independent building foundations.

### Conclusion of the experimental assessment of noise reduction through an anechoic box

Regarding the use of the anechoic box for the recordings included in the InsectSound1000 dataset, in addition to the insect sounds recorded inside the box, it is possible that the dataset contains some low-frequency sounds stemming from the recording environment, which made their way through the box walls. Therefore, the chances of such low-frequency sounds occurring during the recordings for the dataset were minimised by conducting them overnight (see the Recording Process section). Furthermore, to avoid these background sounds creating bias in the dataset, each insect species was recorded multiple nights to reduce the likelihood of similar background sounds occurring in every recording of one species.

Unfortunately, as apparent from the presented sound absorption experiment, it must be assumed that background sounds are present next to the insect sounds in the raw recordings. However, subjectively, there seem to be very few background sounds in the extracted InsectSound1000 sound sample dataset. This can be attributed to, first, the nighttime recordings, and, second, the aggressive high-pass filtering applied during the sample extraction process (see the Data Processing section). Combining the digital high-pass filtering and the very good noise shielding properties of the anechoic box for high frequencies meant that background sounds were very unlikely to trigger sample extraction on their own. The background sounds present in the InsectSound1000 dataset likely occurred at random at the same time as an insect sound signal.

In conclusion, combining physical noise shielding and adapted signal processing allowed for the creation of a dataset that should have adequate labelling accuracy and sound quality to enable automated data processing in machine learning models. The sound and labelling quality of the InsectSound1000 dataset cannot be matched by any other existing insect sound dataset available today^5^ (https://www.tierstimmenarchiv.de).

## Usage Notes

Preliminary experiments with the InsectSound1000 dataset indicate that despite all the precautions taken, some powerful models seem to be able to distinguish the different recording dates in the data of some of the insect species. To prevent data leakage from the training set to the validation and test sets during the training of a machine learning model, the dataset should be split, separating the different recording dates, which means that the models are validated and tested with sounds recorded exclusively on different days than those used for training. A Jupyter Notebook, which splits the dataset into training, validation and test data while ensuring that the recording dates of the three datasets do not overlap, is included in the accommodating GitHub repository (see the code availability section).

If the dataset is not split by recording dates it cannot be ruled out that a model could recognise the recording day in the test data and infer the correct insect class from the recording day instead of learning meaningful properties of the different insect sounds. Given the precautions taken to avoid this problem precisely, such as building an anechoic box and high-pass filtering the signals, the results of these preliminary experiments are astounding. After evaluating the experimental results, there seems to be no common pattern audible to human ears or visible in spectrograms to human eyes that could allow humans to make these distinctions between insect sounds from different recording dates of the same insect. To the human observer, there appear to be no background sounds that could allow this distinction.

In light of these findings, which demonstrate the ability of modern data processing to recognise patterns beyond human perception, it is reasonable to assume that some models might also be able to recognise individual insects. The individual insects used for different recordings in the InsectSound1000 dataset were not documented, and at least for some of the louder insects, such as the *B. terrestris or R. nebulos*, all sounds were recorded from the same individuals.

This finding represents a limitation of the presented Insetsound1000 dataset. When splitting the dataset, the training and test sounds for some of these insects in the dataset might only contain sounds of the same individual insects. Therefore, models trained and tested on only the InsectSound1000 dataset might overfit to recognise these individual insects, meaning that they not only learn to recognise patterns that generally describe the insect species but also the individual insects in particular. This could mean that test scores for models tested on new recordings with different individual insects, even when recorded with the same method, could be a few percent worse than a model evaluation only on data included in the InsectSound1000 dataset. Future work, focusing on only the InsectSound1000 dataset for training and evaluation, should consider these shortcomings of the dataset.

## Data Availability

The Python script used to extract the sound samples collected from the InsectSound1000 dataset from the raw, long-term recordings is available online on the InsetSound1000 GitHub page (https://github.com/Jelt0/InsectSound1000Tools). The script can easily be modified for extraction, e.g., samples of different lengths, different sample rates or even different thresholds for activity detection. Furthermore, the accommodating GitHub repository contains a Jupyter Notebook to split the InsectSound1000 dataset into training, validation and test subsets while ensuring that recording dates do not overlap in the different subsets.
